# Role of Telomeres and Telomerase in Cancer and Aging

**DOI:** 10.3390/ijms24129932

**Published:** 2023-06-09

**Authors:** Gabriele Saretzki

**Affiliations:** Biosciences Institute, Campus for Ageing and Vitality, Newcastle University, Newcastle upon Tyne NE4 5PL, UK; gabriele.saretzki@ncl.ac.uk

Seventeen papers published in 2019 and early 2020 demonstrate the ongoing interest and research concerning telomeres and telomerase in aging and cancer. These include 9 original studies and 8 reviews, displaying a good balance between papers which describe novel molecular mechanisms for telomeres and telomerase, their significance for aging and longevity and the application of novel agents and techniques for the characterisation of or intervention into different types of cancer. Around half of the papers are dedicated to basic biological processes in the area of telomeres and telomerase, such as TERRA transcription, epigenetics, replication stress, stem cell differentiation, mechanisms of mitochondrial protection or the application of CRISPR-Cas (clustered regularly interspaced short palindromic repeats)/Cas9 (CRISPR-Associated Protein 9) techniques. The other half deals with various clinical aspects of the field of telomeres and telomerase, including inhibition of telomerase activity, tumour growth and immunotherapy. Different tumour and disease types have been highlighted, such as paediatric cancer, multiple myeloma, thyroid cancer, breast cancer cell lines, lung disease and cardiovascular disease. In summary, most papers from this edition are focussed on new mechanisms and a better understanding of telomere and telomerase biology with the final aim of developing new and more efficient anti-cancer treatments.

The study of Chang et al. targets aspects of regenerative medicine and emphasises the importance of telomere length (TL) for the differentiation capacity of mouse embryonic stem cells into chondrocytes after somatic cell nuclear transfer, since in *TERC* knock-out mice this property is compromised [1]. Their results confirm the important role of telomerase and telomere length for certain differentiation properties of manufactured stem cells for regenerative purposes.

Le Berre et al. demonstrate that the mechanism of DNA methylation repressing TERRA (telomeric repeat-containing RNA) expression from subtelomeric regions depends on NRF1 binding [2]. While telomeres were previously thought to be purely heterochromatin, it is now accepted that they are also transcribed into long noncoding TERRA molecules from subtelomeric promoters and play an important role in the regulation of telomere biology, DNA repair and telomerase access in both normal and cancer cells [3]. However, there are still many unsolved issues regarding the exact regulatory mechanisms of *TERRA* expression, such as in cancer cells. In particular, the regulatory mechanisms of TERRA via methylation of the subtelomeric CpG island have not been established yet. Thus, the authors of the study set out to address the underlying mechanism which regulates *TERRA* expression through subtelomeric CpG island methylation and used a specific CRISPR/dcas9 (clustered regularly interspaced palindromic repeat/dead CRISPR-associated protein 9) in order to demethylate the CpG islands in Hela cancer cells [2]. This demethylation resulted in higher *TERRA* expression that was dependent on the methyl-sensitive transcription factor NRF1 (nuclear respiratory factor 1). Thus, the study describes a novel mechanism of fine-tuning of TERRA regulation, reinforcing the strong relationship between subtelomeric methylation status and regulation of telomere length by TERRA. These results might have consequences for the treatment of cancer cells in the future, since silencing of TERRA could inhibit telomerase activity and thus be a therapeutic tool against cancer cell proliferation.

Similarly, the review of Yuan and Xu [4] is dedicated to epigenetics, but in this case in relation to the telomerase reverse transcriptase protein (TERT). The authors review the involvement of TERT in epigenetic changes during physiological processes and tumourigenesis, as well as the influence of aberrant epigenetics on the tumour-promoting properties of TERT. This function of the TERT protein is one of the many non-canonical functions of TERT independent of the impact of telomerase activity (which requires both TERT and the telomeric RNA component TERC) on telomeres. Importantly, some of these non-telomeric functions are involved in the development or progression of cancers by promoting cancer cell survival. In a previous study, the authors characterised the causal relationship between *TERT* and *DNMT3B* expression [5]. TERT has also been previously shown to be a transcriptional co-factor that influences gene expression, such as by interacting with the transcription factor Sp1. Since DNMT3 is a target of SP1, the authors speculate about a possible mechanism of how TERT might promote DNA methylation and how both molecules are able to form a signalling network involved in aberrant DNA methylation, AKT activation and other oncogenic activities, resulting in enhanced tumourigenesis [4]. Importantly, TERT-induced epigenetic modifications such as DNA methylation and chromatin remodelling in turn modify *TERT* expression and regulation, forming a positive feedback loop. The authors also discuss the clinical implications of *TERT* promoter hypermethylation as a potential prognostic factor and a potential novel anti-cancer treatment target.

Banszerus et al. analysed the relationship between telomere length (TL) and an epigenetic clock in a human population study [6]. The epigenetic clock employs the weighted DNA methylation fraction as a marker of chronological age. The authors estimated the epigenetic age of around a thousand participants of the LipidCardio Study, a patient cohort with a high prevalence of cardiovascular disease, and compared it to the measured TL in blood cells. It turned out that there was no predictive power in TL, while the epigenetic methylation pattern better described the chronological age of the participants [6]. The authors state that both parameters represent different aspects of aging. It would also be interesting to examine whether TL or DNA methylation are able to predict disease risk in this study cohort. 

Another aspect of telomere biology was reviewed by Veverka and co-workers, who described the mechanism of how the telomeric shelterin complex influences and recruits telomerase to telomeres [7]. Due to the high importance of telomere maintenance and capping by telomerase in cancer cells, the authors emphasise telomerase access to telomeres as a potential target in anti-tumour therapies. They review quantitative assessment and novel structural information for new approaches of telomerase inhibition. Shelterin forms a protective protein assembly around telomeric DNA and is involved in many processes related to telomere length regulation, including access of telomerase to telomeres. The ongoing access to telomeres for maintenance and capping is essential for tumour cells in order to ensure continuous cell proliferation. Several shelterin proteins, such as TPP1 and Pot1, but also TRF1 and TRF2, are responsible for opening the t-loop for access of telomerase to the G-rich single-stranded telomeric 3′overhang. The authors studied the interactions of telomeric proteins (shelterin) and telomeric DNA, as well as within the shelterin complex itself, and identified TPP1 as the limiting component for shelterin assembly and for telomerase recruitment. For the purpose of inhibiting telomerase biological activity on telomeres, a block of these interactions could facilitate a lower tumour cell proliferation and TPP1 could be a promising target here.

Shekhidem et al. investigated telomeres in the context of aging and longevity and published a study analysing the relationship between telomeres and longevity considering age-related differences in telomere shortening between short- and long-lived rodents [8]. They cross-sectionally examined average telomere length correlated to age and found that of two long-lived species, the naked mole rat (NMR) has no measurable telomere shortening over its rather long life (around 30 years), while the other species—the blind mole rat *Spalax* (with a mean lifespan of around 20 years)—displayed the same age-related telomere shortening rate as mice and rats (2–3 years) with short lifespans [8]. This result implicates that NMRs are an exception within long-lived species (rodents), but that this mechanism is not the rule for longevity. Other studies have shown previously that long-lived species seem to have shorter telomere shortening kinetics than short-lived ones and might have better telomere maintenance mechanisms, such as higher telomerase activity, or be better protected from environmental or endogenous oxidative stresses, which are known to be involved in the modulation of telomere shortening rates [9]. Although it seems that NMRs have developed specific mechanisms of telomere maintenance during their entire lifespan and it is tempting to speculate that they have increased telomerase activity (TA), to my knowledge this has not yet been experimentally demonstrated and is thus a topic for future research.

Another interesting paper, from Billard and Poncet, is dedicated to the aging aspect, reviewing the implications of replication stress on telomeric and mitochondrial DNA [10]. This process is important since cellular senescence is characterised as a stress response, resulting in an irreversible cell cycle arrest in dividing cells. It is a rather novel aspect in aging research that replication stress in telomeric and mitochondrial DNA shares various aspects, such as G-quadruplexes, D-loops, RNA:DNA heteroduplexes, epigenetic marks and supercoiling, as potential causes for senescence and aging. Interestingly, both cellular compartments use similar mechanisms to counteract replication stress, such as endonucleases, topoisomerases, helicases and primases. Moreover, specific telomeric proteins such as TIN2 and TERT are able to shuttle to mitochondria and modulate mitochondrial metabolism and ROS levels there [11]. It is important to mention that telomeres induce senescence not only via a critical shortening of length but to a large extent also by accumulating DNA damage independent of length or presence of telomerase activity [12,13]. This specifically applies to postmitotic cells that do not shorten their telomeres. This DNA damage can originate from events such as replication stress, as well as increased oxidative stress, to which telomeres are highly sensitive [9]. The authors specify the causes and results for replication stress in telomeres and mitochondria in great detail and review the available literature carefully and comprehensively.

Similarly, mitochondrial DNA homeostasis via TERT localisation was studied by Martens et al. [14]. The authors demonstrate that the TERT protein that shuttles from the nucleus to mitochondria upon oxidative stress [11] does not influence mitochondrial DNA repair (base excision repair, BER) directly, but instead even without shuttling specifically increases the protein amount of mitochondrial superoxide dismutase (MnSOD). This is a primary antioxidant that detoxifies mitochondrially generated superoxide and transforms it into less toxic hydrogen peroxide, which can also leave the mitochondria and damage other cellular macromolecules such as nuclear DNA. In contrast, other antioxidants, such as cytoplasmic Cu/Zn SOD (SOD1) or catalase, were not increased under the given conditions [14]. Furthermore, upstream components of MnSOD such as the transcription factor Foxo3a were also increased in fibroblasts over-expressing telomerase/TERT. Intriguingly, both MnSOD and FOXO3a were increased under serum-free medium conditions indistinguishable from conditions of higher oxidative stress, which resulted in shuttling of the TERT protein out of the nucleus into mitochondria while this process was not induced under serum-free conditions [14]. Consequently, TERT/telomerase seems not to have a direct physical influence on MnSOD or mitochondrial metabolism but rather influences the protein via indirect signalling mechanisms; for example, via FOXO signalling, which is increased under serum-free conditions or other pathways that were not analysed.

Arish et al. summarised the literature on the role of telomeres and telomerase for the prognosis and treatment of interstitial lung diseases (ILDs), including idiopathic pulmonary fibrosis (IPF) as well as its associated pulmonary pathologies [15]. While it is accepted that DNA damage plays an important role in these diseases, the authors aimed to better understand the role of telomeres as well. A clinical association between telomerase and lung disease was found more than 20 years ago in dyskeratosis congenita, which is often related to mutations of telomerase genes or dyskerin (DKC), resulting in shorter TL [16]. Similar mutations also cause pulmonary fibrosis. In contrast to such inherited diseases, IPF is age-related, with short telomeres as a risk factor. Importantly, mutations in telomerase genes as well as short telomeres are able to influence the disease course of patients and often have a worse prognosis, while certain types of treatment are not indicated in such cases. Thus, short telomeres in a screening approach might be useful to preferentially select these cases for lung transplantation in ILD, and the authors recommend such testing for TL in order to identify more risk factors for ILDs and in order to develop better treatment options in the future. Interventional studies using telomerase activators such as GRN510 were able to improve experimental (bleomycin-induced) lung IPF, decreasing the amount of senescence, presumably by increasing TL via TA [17].

Morcos et al. analysed the telomeric shelterin protein TERF1/Pin2 and found that a new splice version of it is downregulated specifically in testis-derived cancer cells [18]. TERF1 is involved in telomere length regulation with a specific role in the S-phase during replication fork movement. While TERF1 overexpression reduces telomere length by inhibiting telomerase-dependent elongation, loss of it increases the fraction of fragile telomeres in the S-phase [19]. The new splice version possesses a 30-amino-acid internal insertion near to the C-terminus of TERF1 and is preferentially expressed in human spermatogonial and hematopoietic stem cells, and thus is strongly tissue-specific and was named TERF-tsi. In contrast, primary human cells or established cancer cell lines did not express this factor [18]. The authors demonstrated that while normal TERF1 and its splice variant PIN2 persisted during testis-derived tumourigenesis, TERF-tsi was specifically downregulated in this process, suggesting a function as a tumour suppressor for this new splice version.

The review of Ackermann and Fischer summarises the current limited understanding of the role of telomere length in paediatric cancer [20]. At the same time, the authors describe recent advances in the molecular characterization of structure and function of telomeres, as well as the regulation of telomerase activity in cancer pathogenesis with a special emphasis on telomere maintenance in childhood neuroblastoma. As known for adult cancer types, a new study showed that paediatric cancers also seem to have shorter telomeres than normal tissue [21]. To keep these telomeres capped and functional, most cancer cells activate high levels of telomerase, which are often associated with *TERT* promoter mutations. In contrast, those have not yet been described in paediatric cancers, which represent only 1% of all cancers. Regarding telomere biology, childhood neuroblastoma have been best investigated in the past. Here, either the transcription factor for *TERT*, MYCN was upregulated or there are genomic rearrangements of the *TERT* locus, as recently discovered. Such tumours with high levels of telomerase activity had, comparable to adult cancers, short telomeres, while other cancer types activated the alternative telomere lengthening mechanism (ALT) with longer telomeres. Various studies agreed that tumours with an activated telomere maintenance mechanism (TMM) have a more aggressive nature. In contrast, tumours without such a mechanism did not reach cell immortality and thus tended to spontaneously regress. The authors also describe various other childhood tumour types and conclude that a better understanding of telomere biology and TMMs can be used for diagnosis, risk estimation and improved treatment strategies of these paediatric tumours. 

A similar review on the role of telomeres and telomerase was written by Donati and Ciarrocchi and focused on thyroid cancer (TC) [22]. This cancer type displays a highly aggressive behaviour, but is also accessible for curation. In contrast, cancers with metastases have a poor prognosis and often lack therapeutic options. In these tumours, TERT promoter mutations create novel binding sites for transcription factors, resulting in increased TERT expression and telomerase activity. Another mechanism is *TERT* gene amplification. The authors summarise the current knowledge about telomere regulation in TCs, considering both canonical and non-telomeric functions of telomerase and TERT. They also discuss the association between telomere homeostasis and cancer responses to therapy; for example, with telomerase inhibitors and epigenetic modifiers. 

In addition to telomerase inhibitors which so far have not proven to be clinically successful, other approaches use telomerase/TERT-targeting cancer immunotherapy, which is the topic of a review by Mizukoshi and Kaneko [23]. The authors describe this technique as a possible paradigm change, since it mainly targets the host’s immune system in order to enable it to fight cancer cells. Cancer immunotherapy is an alternative treatment to surgery, chemo- or radiation therapy, and involves using cytokines, antibodies and checkpoint inhibitors, as well as immune cells, such as dendritic cells (DCs) and T cells. Due to the enormous significance of telomerase and its antigen TERT for the immortality of tumour cells, its broad appearance in around 85% of tumours, as well as its high significance for the function of cancer stem cells, TERT has been identified it as a potential target for tumour therapies. Consequently, a large number of hTERT-derived peptides have been identified as targets for cancer immunotherapy and various vaccines based on hTERT-derived peptides have been evaluated for different types of cancer. The authors describe details of several such vaccines that as a majority are expressed on MHC class I molecules, enhancing the response of cytotoxic T-cells (CTL), while others are expressed on MHC class II molecules and predominantly induce T_helper_ cell responses. While several of these anti-hTERT immunotherapies have been recently developed and some have been clinically implemented, there is still a need to combine them together in multiplex immunotherapies or with other forms of therapy, such as chemotherapy, in order to achieve the best benefits for tumour patients.

The remaining four papers describe different approaches of targeting the TERT component of telomerase. Shalem-Cohavi et al. demonstrate the use of proteasomal inhibitors on telomerase activity and it’s regulation in multiple myeloma (MM) cells [24]. While these inhibitors are already used in the therapy of MMs, their effect on telomerase activity has not been thoroughly investigated previously. The group had previously shown the effect of the inhibitor bortezomid on telomerase inhibition, and in this study they investigated the effect of the new inhibitors MG-132 and epoxomicin (EP) on different MM cell lines and found differential effects of both inhibitors and cell lines regarding the downregulation of telomerase and cell proliferation. More mechanistic studies were performed on the stronger-acting EP and the authors found both transcriptional and post-transcriptional regulation of hTERT, since EP in one MM cell type decreased the expression and binding of various transcription factors (TF) to the *hTERT* promoter. The authors conclude that TERT is a novel target of proteasomal inhibitors, which could lead to novel therapeutic approaches.

With a similar aim, Konieczna et al. analysed the effect of the telomerase inhibitor TMPyT4 on the adhesion and migration behaviour of two common breast cancer cell lines [25]. Telomeres are known to form quadruplex structures which are often associated with oncogenic or tumour-suppressor proteins. TMPyT4 is a cationic porphyrin that stabilises these quadruplexes and apparently inhibits telomerase, presumably by preventing the access to telomeres. The authors found that TMPyT4 treatment decreased cell viability, as well as migration and adhesion of the two analysed breast cancer cell lines MCF-7 and MDA-MB-231, and was dose-dependent at 48 and 72 h of treatment, possibly impacting on tumour cell behaviour and decreasing their survival and spreading. However, the authors emphasise that the detected effects were most likely independent of telomeres and telomerase, although a decrease in *hTERT* expression, protein level and telomerase activity was demonstrated at 72 h, but this might be attributed to decreased cell viability. Interestingly, no DNA damage was apparently inflicted during TMPyT4 treatment, which is puzzling considering the decline in cell viability which corresponded to a downregulation of cell-cycle-related proteins, but no increased apoptosis was found in MDA-MB-231 cells [25]. Thus, more mechanistic studies are required to fully understand the possible effects of TMPyT4 on tumour cells.

A very elegant way of genetically disrupting the *hTERT* gene in cancer cells using CRISPR/Cas9 was demonstrated by Wen et al. [26]. As expected, this disruption of exon 4 of one TERT allele in HeLa cells resulted in a strong decrease of telomerase activity as well as cancer cell proliferation due to increased apoptosis. However, the authors did not analyse telomere length, which means they cannot draw conclusions on whether the effects target canonical or non-canonical pathways of TERT/telomerase, which are both able to compromise cancer cell survival. Importantly, in vivo experiments in the study demonstrated that *TERT* haplo-insufficient tumour cells failed to form xenografts after transplantation into nude mice. Consequently, such a gene-editing-mediated *TERT* knockout could be an attractive novel therapeutic option for cancer therapy in the future. 

The final paper, by Lee et al., reviews the role of cellular senescence in the context of tumours and describes the role of stilbene compounds such as resveratrol which inhibit tumour growth by inducing cellular senescence and inhibiting telomerase activity and recommend them as senescence inducers for use as future cancer treatments [27]. This strategy is based on the fact that senescence acts as a tumour suppressor and is thus interesting for tumour therapies [28]. Agents such as BIBR1532 bind to hTERT, thereby inhibiting TA, shortening TL and finally inducing cellular senescence, as has been demonstrated in many different cancer types previously [29]. 

Figure 1 summarises the topics of the Special Issue regarding their focus on telomeres and telomerase for aging and cancer. 

## Figures and Tables

**Figure 1:**
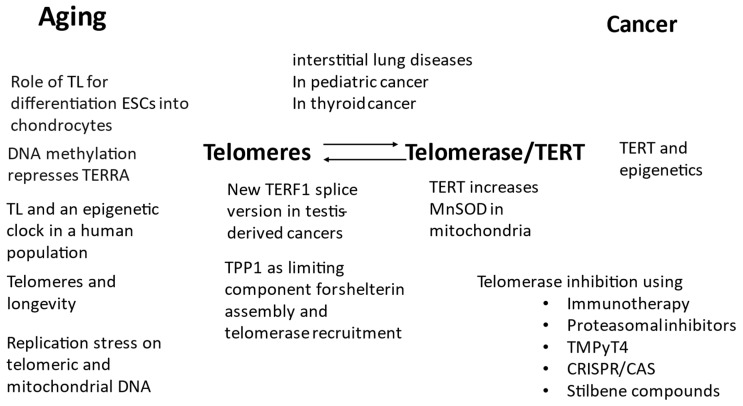
Overview of the 17 published papers and their main topics regarding telomeres and telomerase/TERT in aging and cancer. TL: telomere length, ESC: embryonal stem cells.
